# Supplementary material for: Exploring the complex free-energy landscape of the simplest glass by rheology
Source: Nat Commun. 2017 Apr 11;8:14935. doi: 10.1038/ncomms14935 (PMC5394243; doi:10.1038/ncomms14935)
Supplement: Supplementary Information — Supplementary Figures, Supplementary Notes, Supplementary Methods and Supplementary References [file ncomms14935-s1.pdf]

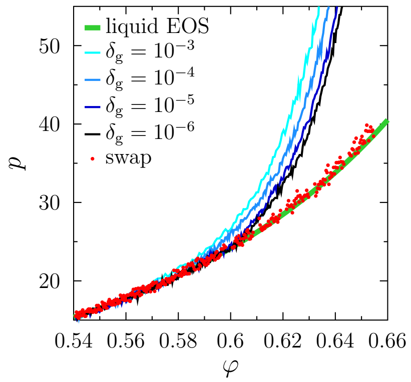

Supplementary Figure 1. Evolution of the reduced pressure p under compression, for a system of $N = 2000$ particles. The data obtained from the swap algorithm agree with the Carnahan-Stirling empirical liquid EOS Eq. (Supplementary Equation 4) [1]. For comparison, we also plot data obtained from pure compression done by the LS algorithm without the swap for a few different compression rate δ_g. The swap algorithm falls out of equilibrium at much higher φ, compared to the standard compressions.

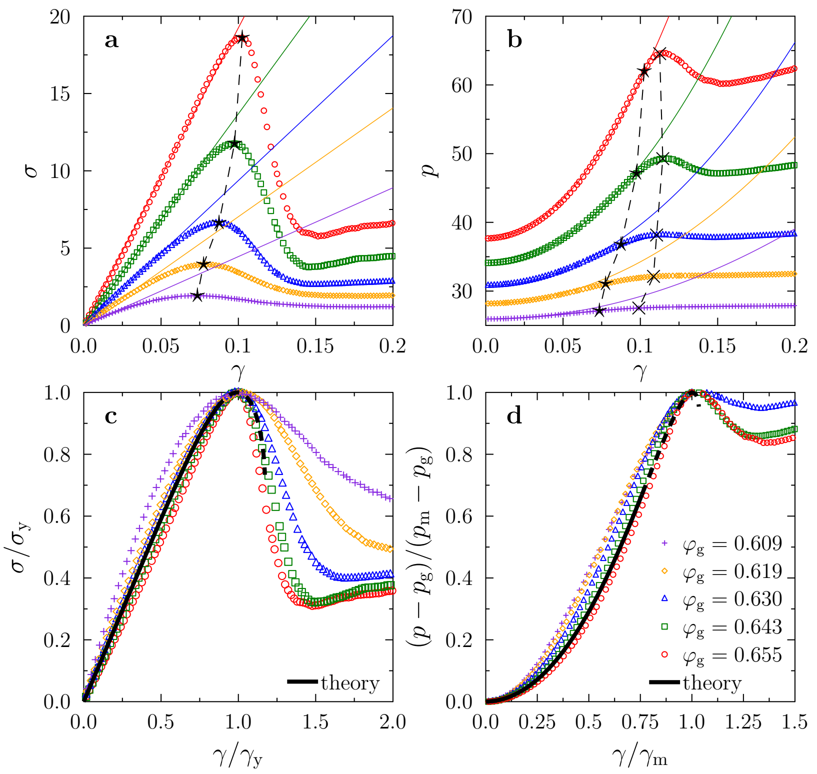

Supplementary Figure 2. Quasi-static shear on equilibrium configurations prepared at a few different φ_g. The systems consist of $N = 1000$ particles, and the data are averaged over $N_s = 100$ samples and $N_{th} = 10 - 20$ realizations for each sample. (a) The shear stress σ and (b) the pressure p are plotted as functions of the shear strain γ. At small γ, the stress-strain curve is fitted to a linear function $\sigma = \mu\gamma$ (lines), and the pressure-strain curve is fitted to a quadratic function $p = p_g + R\gamma^2$ (lines). The star marks the peak of the stress-strain curve, which represents the yielding point (γ_y, σ_y), and the cross marks the peak (γ_m, p_m) of the pressure-strain curve. The parameters μ, γ_y, σ_y, γ_m, p_m, and R are reported in Supplementary Figure 3 as functions of φ_g. (c) The rescaled stress-strain curves and (d) the rescaled pressure-strain curves are compared to the mean-field theoretical predictions (black line) [2], for the equilibrium volume fraction $\hat{\varphi}_g = 2^d\varphi_g/d = 7$, where the dimension $d = 3$. Here the solid line part is the stable 1-step replica symmetry breaking (1RSB) solution, and the dashed line part is the unstable 1RSB solution [2]. We have checked that the theoretical results are insensitive to $\hat{\varphi}_g$ on these rescaled plots.

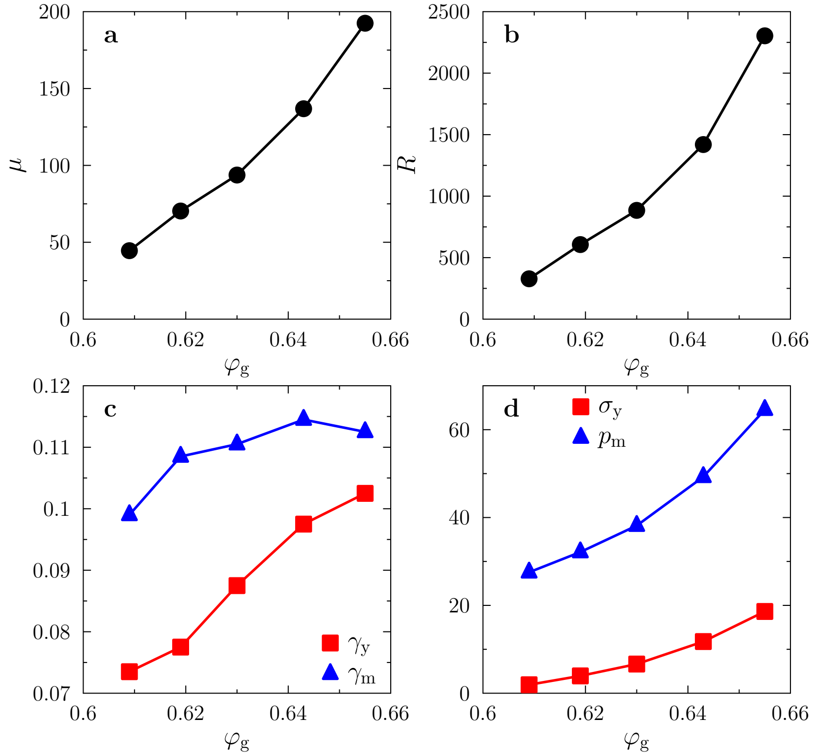

Supplementary Figure 3. Elastic and yielding parameters of equilibrium configurations. We plot as functions of $\varphi_{\rm g}$, (a) the shear modulus μ, (b) the dilatancy parameter R, (c) the yield strain $\gamma_{\rm y}$ and the strain $\gamma_{\rm m}$ at the maximum pressure in the pressure-strain curve, and (d) the yield stress $\sigma_{\rm y}$ and the maximum pressure $p_{\rm m}$.

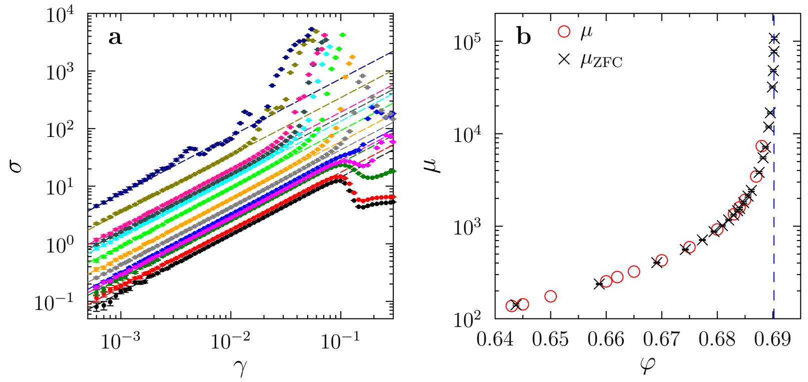

Supplementary Figure 4. Quasi-static shear on out-of-equilibrium glass states. The states are compressed from $\varphi_{\rm g} = 0.643$ to target density φ before the shear is applied. The systems consist of $N = 1000$ particles, and the data are averaged over $N_{\rm s} = 100$ samples and $N_{\rm th} = 10-30$ realizations for each sample. (a) The shear stress σ as a function of γ, for a few different φ (from bottom to top, $\varphi = 0.645, 0.65, 0.66, 0.662, 0.665, 0.67, 0.675, 0.68, 0.683, 0.684, 0.685, 0.687, 0.688$), where $\varphi_{\rm G} = 0.684(1)$. The linear response regime is fitted to $\sigma = \mu\gamma$ (lines). (b) The shear modulus μ obtained from this fitting is compared to the ZFC shear modulus $\mu_{\rm ZFC}$ (we use $\gamma = 2 \times 10^{-3}$, see the main text), both of which diverge approaching to the jamming limit $\varphi_{\rm J} = 0.690(1)$ (vertical dashed line). For all figures, the error bars denote the standard error of the mean.

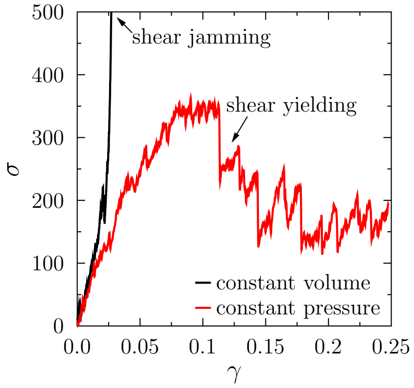

Supplementary Figure 5. Quasi-static shear on the same glass state ($\varphi_g = 0.643$, $\varphi = 0.688$, and $N = 1000$) under constant volume and constant pressure show different behaviors at large γ. We observe shear jamming in the constant volume simulation and shear yielding in the constant pressure simulation.

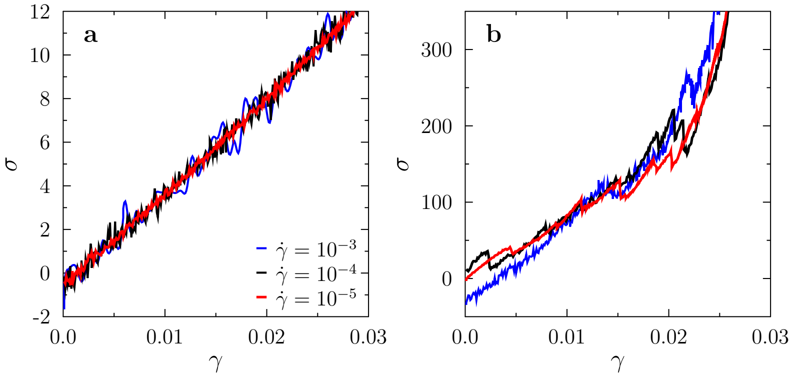

Supplementary Figure 6. The stress-strain curves of three *different realizations* of the compressed glass at (a) $\varphi = 0.670$, and (b) $\varphi = 0.688$, obtained from the *same equilibrated sample* of $N = 1000$ particles at $\varphi_g = 0.643$. They are driven by *different strain rates* $\dot{\gamma}$ as indicated by the legend.

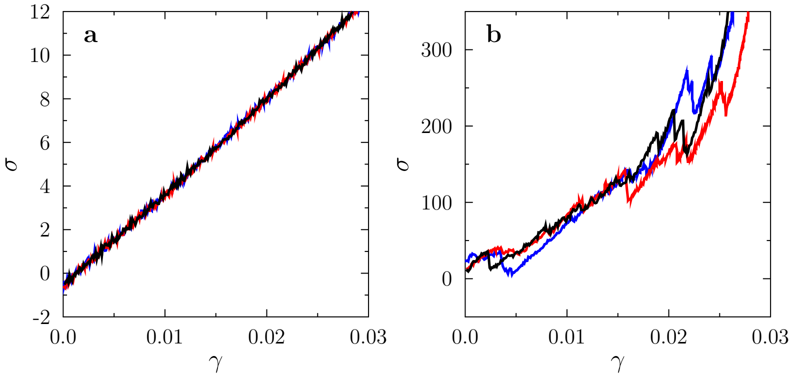

Supplementary Figure 7. Stress-strain curves of three *different realizations* of the compressed glass at (a) $\varphi = 0.670$, and (b) $\varphi = 0.688$, obtained from the *same equilibrated sample* of $N = 1000$ particles at $\varphi_g = 0.643$. They are driven by the *common strain rates* $\dot{\gamma} = 5 \times 10^{-6}$ for $\varphi = 0.670$, and $\dot{\gamma} = 10^{-4}$ for $\varphi = 0.688$.

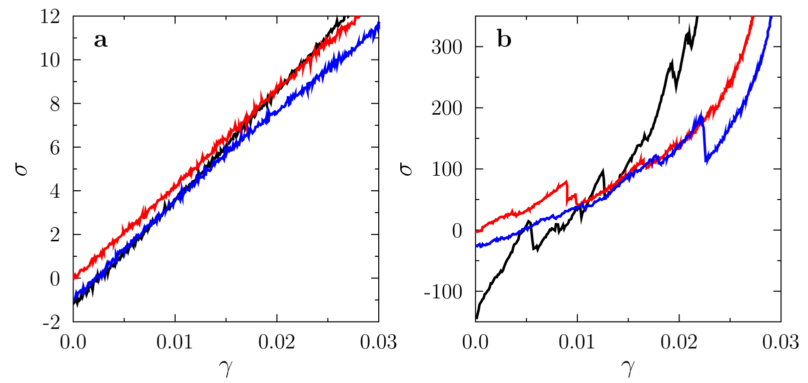

Supplementary Figure 8. Stress-strain curves on the compressed glasses at (a) $\varphi = 0.670$, and (b) $\varphi = 0.688$, obtained from three *different equilibrated samples* of $N = 1000$ particles at $\varphi_{\rm g} = 0.643$. They are driven by the *common strain rates* $\dot{\gamma} = 5 \times 10^{-6}$ for $\varphi = 0.670$, and $\dot{\gamma} = 10^{-4}$ for $\varphi = 0.688$.

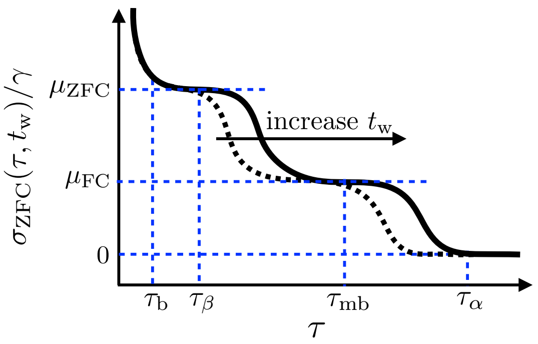

Supplementary Figure 9. Schematic illustration of the relaxation of ZFC shear stress $\sigma_{\rm ZFC}(\tau, t_{\rm w})$ after an instantaneous shear strain γ is applied, in the Gardner phase $\varphi > \varphi_{\rm G}$ [3]. The two shear moduli $\mu_{\rm ZFC}$ and $\mu_{\rm FC}$ correspond to the first and second plateaus respectively. Corresponding time scales for the black solid line are indicated. The dotted black line represents a shorter waiting time $t_{\rm w}$. See also Fig. 2 of [3].

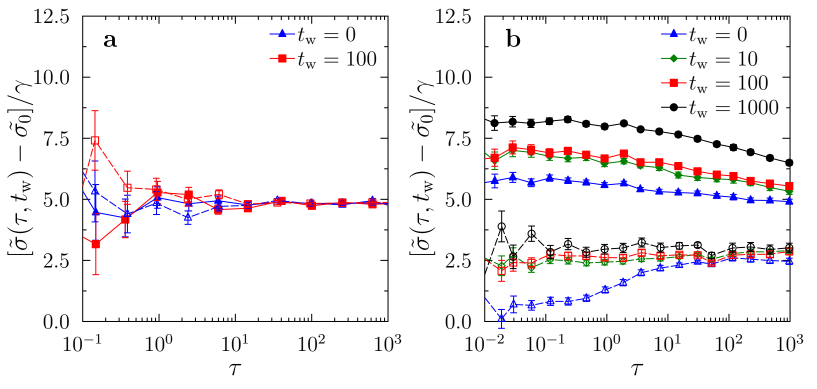

Supplementary Figure 10. Relaxation of $\tilde{\sigma}_{\rm ZFC} = \sigma_{\rm ZFC}/p$ (filled symbols) and $\tilde{\sigma}_{\rm FC} = \sigma_{\rm FC}/p$ (open symbols) at (a) $\varphi = 0.670$ and (b) $\varphi = 0.688$, for a few different $t_{\rm w}$, under a quasi-static shear strain $\gamma = 10^{-3}$. The system consists of $N = 1000$ particles, and is compressed from $\varphi_{\rm g} = 0.643$. The data are obtained for one individual sample, but averaged over $N_{\rm th} \sim 1000$ independent realizations of the compressed glass. The remanent stress $\tilde{\sigma}_0$ has been subtracted from $\tilde{\sigma}$.

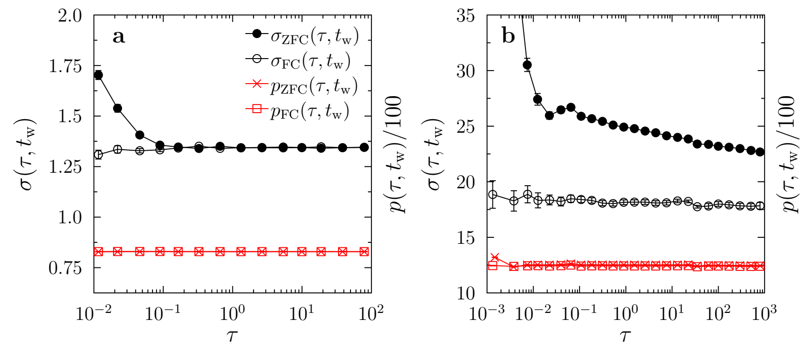

Supplementary Figure 11. Time evolution of the ZFC pressure $p_{\rm ZFC}(\tau, t_{\rm w})$ and the FC pressure $p_{\rm FC}(\tau, t_{\rm w})$ after an instantaneous increment of shear strain $\gamma = 10^{-3}$, at (a) $\varphi = 0.670$ and (b) $\varphi = 0.688$. For comparison, we also display the behaviour of the shear stress in the ZFC and FC protocols. Data are averaged over many realizations of compressed glasses obtained from a single equilibrated sample at $\varphi_{\rm g} = 0.643$ with $N = 1000$ particles. The pressure values are rescaled by a factor of 1/100. This plot shall be compared with Fig. 2 in the main text (only $t_{\rm w} = 1000$ data are shown).

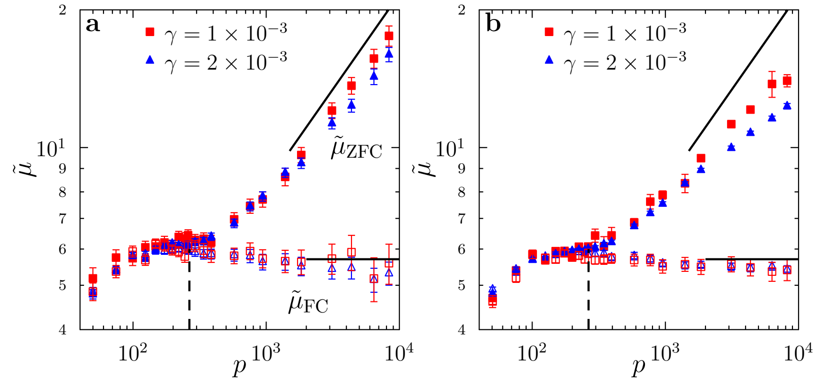

Supplementary Figure 12. γ-dependence on the ZFC and FC shear moduli. The data are obtained for (a) $N = 500$ and (b) $N = 2000$ particles, and are averaged over $N_{\rm s} \approx 200$ samples and $N_{\rm r} \approx 100$ individual realizations for each sample. The vertical dashed line represents the Gardner transition [1], and the solid lines are the mean-field predictions $\mu_{\rm ZFC} \sim p^{1.41574}$ and $\mu_{\rm FC} \sim p$ [3].

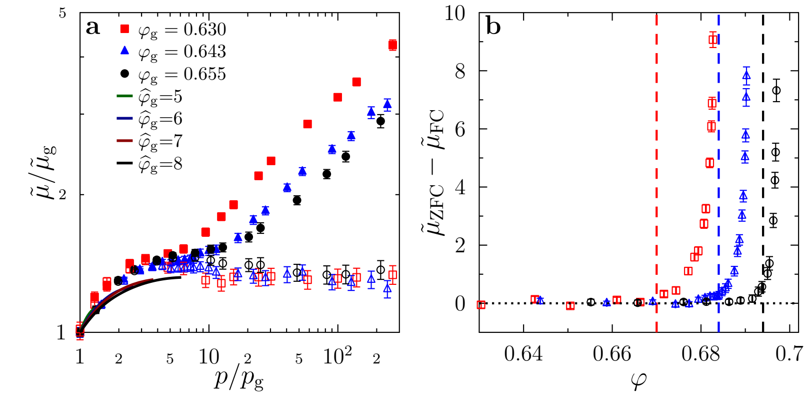

Supplementary Figure 13. $\varphi_{\rm g}$-dependence on the ZFC and FC shear moduli. The data are obtained for $N = 1000$ particles, and are averaged over $N_{\rm s} \approx 200$ samples and $N_{\rm r} \approx 100$ individual realizations for each sample. To compute the shear modulus, $\gamma = 2 \times 10^{-3}$ is used. (a) The numerical data of the rescaled ZFC shear modulus $\tilde{\mu}_{\rm ZFC} = \mu_{\rm ZFC}/p$ (filled symbols) and the rescaled FC shear modulus $\tilde{\mu}_{\rm FC} = \mu_{\rm FC}/p$ (open symbols) with a few different $\varphi_{\rm g}$, are compared to the mean-field theory (lines) with different $\hat{\varphi}_{\rm g}$, where $\hat{\varphi}_{\rm g} = 2^d \varphi_{\rm g}/d$ with $d = 3$, following the convention used in Ref. [2]. Both numerical and theoretical results are rescaled by the reference values $\tilde{\mu}_{\rm g} = \mu_{\rm g}/p_{\rm g}$ and $p_{\rm g}$ at $\varphi_{\rm g}$. (b) The difference $\tilde{\mu}_{\rm ZFC} - \tilde{\mu}_{\rm FC}$ as a function of φ for a few different $\varphi_{\rm g}$, where the Gardner transitions $\varphi_{\rm G}$ (values from Ref. [1]) are marked by vertical lines.

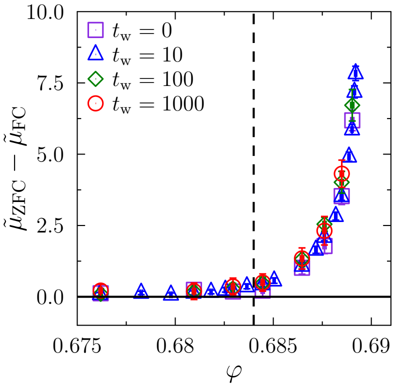

Supplementary Figure 14. The difference $\tilde{\mu}_{\rm ZFC} - \tilde{\mu}_{\rm FC}$ is plotted as a function of φ, for $\varphi_{\rm g} = 0.643$ and $N = 1000$. The shear moduli are measured at $\tau = 1$ for a few different waiting time $t_{\rm w}$. The data are obtained by using $\gamma = 2 \times 10^{-3}$, and are averaged over $N_{\rm s} \approx 100$ samples and $N_{\rm r} \approx 50$ independent realizations for each sample. The vertical dashed line represents $\varphi_{\rm G} = 0.684$ [1].

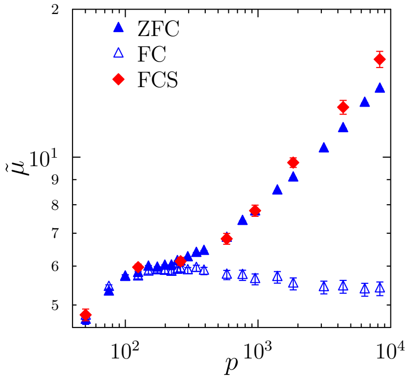

Supplementary Figure 15. The reduced shear modulus obtained from the third protocol (FCS) is compared to $\tilde{\mu}_{\rm ZFC}$ and $\tilde{\mu}_{\rm FC}$, for $\varphi_{\rm g} = 0.643$, $N = 1000$, and $\gamma = 2 \times 10^{-3}$. The data are averaged over $N_{\rm s} \approx 200$ samples and $N_{\rm r} \approx 100$ individual realizations for each sample.

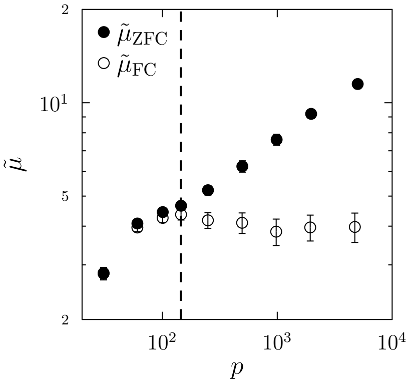

Supplementary Figure 16. Protocol-dependent shear modulus of a bidisperse hard disk glass former, where the vertical dashed line marks the Gardner transition estimated independently in Ref. [1]. The data are obtained for $\varphi_{\rm g} = 0.808$, and are averaged over $N_{\rm s} \approx 100$ samples and $N_{\rm th} \approx 100$ realizations for each sample.

SUPPLEMENTARY NOTE 1 – MODEL AND OBSERVABLES

Polydisperse hard sphere system

We study an assembly of N polydisperse HSs whose diameters are distributed according to a probability distribution [1, 4],

$$P(D) \sim D^{-3}, \qquad D_{\rm min} \le D < D_{\rm min}/0.45. \qquad \text{(Supplementary Equation 1)}$$

This distribution is chosen to optimize the swap algorithm so that denser equilibrium configurations can be obtained, while ensuring that crystallization is suppressed [4]. The control parameter is the number density $\rho = N/V$ or the volume fraction $\varphi = (\pi/6)\rho \int_{D_{\rm min}}^{D_{\rm min}/0.45} dD P(D) D^3$, where V is the volume of the system. The mode-coupling theory (MCT) dynamical crossover density is $\varphi_{\rm d} = 0.594(1)$ [1]. The simulation time t is expressed in units of $\sqrt{\beta m \bar{D}^2}$, where the inverse temperature β, the particle mass m, and the mean particle diameter $\bar{D} = \int_{D_{\rm min}}^{D_{\rm min}/0.45} dD P(D) D$ all set to unity.

Shear stress and pressure

For a HS system, the stress is entropic. The stress tensor is given by

$$\Sigma_{mn} = -\frac{1}{V} \sum_{i<j} \mathbf{r}_{ij,m} \mathbf{f}_{ij,n} \qquad \text{(Supplementary Equation 2)}$$

where $\mathbf{r}_{ij,m}$ is the m-th component of the separation vector $\mathbf{r}_{ij} = \mathbf{r}_i - \mathbf{r}_j$ between particles i and j, and $\mathbf{f}_{ij,n}$ is the n-th component of the inter-particle force $\mathbf{f}_{ij}$. The force $\mathbf{f}_{ij}$ is computed from the exchange rate of the momentum between i and j. In our shear protocols, we are interested in the z-x element of the stress tensor (we omit the subscript),

$$\Sigma = -\frac{1}{V} \sum_{i<j} z_{ij} \mathbf{f}_{ij,x}. \qquad \text{(Supplementary Equation 3)}$$

The pressure P is the negative average of three diagonal elements of the stress tensor, i.e., $P = -(\Sigma_{xx} + \Sigma_{yy} + \Sigma_{zz})/3 = \frac{1}{3V} \sum_{i<j} \mathbf{r}_{ij} \cdot \mathbf{f}_{ij}$. In this study, we report results in the units of reduced pressure $p = \beta P/\rho$ and reduced stress $\sigma = \beta \Sigma/\rho$.

SUPPLEMENTARY NOTE 2 – QUASI-STATIC SHEAR ON EQUILIBRIUM CONFIGURATIONS

First let us report data obtained by quasi-static shear on *equilibrium* configurations at a few different $\varphi_{\rm g}$ (Supplementary Figure 2). As we noted in the main text, the system is in the liquid state in the thermodynamic sense (the Kauzmann density $\varphi_{\rm K}$, if any, is larger then $\varphi_{\rm g}$), but the α-relaxation time is much larger than our simulation time scales so that the system behaves as a solid. The stress-strain curve, averaged over many samples and realizations, shows a linear elastic regime at small γ, followed by yielding. We define the location of the peak in the stress-strain curve as the yield strain $\gamma_{\rm y}$. Note that the definition of $\gamma_{\rm y}$ is more ambiguous for the stress-strain curve of a single realization from a single sample (for example, see Fig. 1b in the main text). In this study, we do not attempt to precisely determine $\gamma_{\rm y}$ for each single stress-strain curve. The shear modulus μ is determined by $\mu = \sigma/\gamma$ in the elastic regime. Both $\gamma_{\rm y}$ and μ grow with $\varphi_{\rm g}$ (Supplementary Figure 3). The yield strain $\gamma_{\rm y}$ and the yield stress $\sigma_{\rm y}$ (which is the stress at $\gamma_{\rm y}$) appear to vanish continuously around $\varphi_{\rm g} \approx \varphi_{\rm d}$. On the other hand, the shear modulus μ appears to remain finite at $\varphi_{\rm d}$, which implies a discontinuous jump of μ at $\varphi_{\rm d}$ being consistent with the mean-field theory [3]. In the elastic regime, the dilatancy effect is observed: the pressure p increases quadratically with γ, i.e., $p = p_{\rm g} + R\gamma^2$, where $p_{\rm g}$ is the pressure at $\varphi_{\rm g}$ and $\gamma = 0$, and R is the dilatancy parameter. The onset of the peak in the pressure-strain curves lags behind the yielding, i. e. the peak of the stress-strain curves. We compare our numerical data to the mean-field theoretical prediction [2], and find reasonable agreement on rescaled plots as shown in Supplementary Figure 2.

SUPPLEMENTARY NOTE 3 – QUASI-STATIC SHEAR ON OUT-OF-EQUILIBRIUM CONFIGURATIONS

Stress-strain curves

Next, let us present quasi-static shear data of *out-of-equilibrium* configurations. These configurations are obtained by compressing the equilibrium configurations from $\varphi_{\rm g}$ to a target density φ, at a constant compression rate $\delta_{\rm g} = 10^{-3}$. Supplementary Figure 4a shows that at small γ, the average stress-strain curve has a linear regime, which shrinks with increasing φ. Note that the data presented here are obtained by averaging over many samples and realizations, while the data in Fig. 1 (main text) are for one single sample and one single realization. For $\varphi > \varphi_{\rm G}$, the apparent linear regime in Supplementary Figure 4a is not truly elastic, since it is averaged over many mesoscopic plastic events (MPEs) (Fig. 1). Thus the shear modulus defined as $\mu = \sigma/\gamma$ in the linear regime is not only due to purely harmonic responses but also involves non-affine corrections due to the plastic events. With this point being clarified, we show that the shear modulus μ obtained from fitting the data in the linear regime, is basically consistent with $\mu_{\rm ZFC}$ presented in the main text (Supplementary Figure 4b).

At larger γ, we find that with increasing φ, the shear yielding disappears and the shear jamming emerges (Supplementary Figure 4a), which can be also observed in Fig. 1. Note that the simulation is performed under the constant volume condition. If we instead fix the pressure and allow the volume to change, then the shear jamming does not appear and the shear yielding exists, even at large φ (Supplementary Figure 5). We also stress that the shift from the shear yielding to the shear jamming is not correlated to the Gardner transition. In fact, Supplementary Figure 5 shows that it is possible to observe both MPEs (at small γ) and yielding (at large γ) in the same stress-strain curve. The key difference is that, after a MPE, the system remains in the same basin although it escapes from the sub-basin, and therefore it still behaves like a solid, while after yielding, the system escapes from the basin and essentially behaves like a fluid.

We next discuss in detail how the measurements of stress-strain curves depend on factors such as the compression rate $\delta_{\rm g}$, the shear rate $\dot{\gamma}$ (Supplementary Figure 6), the realization (Supplementary Figure 7), and the sample (Supplementary Figure 8). First of all, although these configurations are in principle out-of-equilibrium, they reach *restricted equilibrium* [2] for $\varphi < \varphi_{\rm G}$, i.e., they are nearly equilibrated within their glass basins. As shown in Ref. [1], neither structural relaxation nor aging can be observed within the simulation time scale. According to that, in this regime, the results presented here should be nearly unchanged if a slower compression rate is used. The situation is different for $\varphi > \varphi_{\rm G}$: because the time scale diverges in this regime, it is difficult to obtain even the restricted equilibrium configurations and the data would be $\delta_{\rm g}$-dependent. Effectively, decreasing $\delta_{\rm g}$ is equivalent to increasing the waiting time $t_{\rm w}$. Since the $\delta_{\rm g}$-dependence has been well studied in previous work [1], we do not repeat the analysis here. For other factors, in the regime $\varphi < \varphi_{\rm G}$, our results are independent of the shear rate (Supplementary Figure 6) and realizations (Supplementary Figure 7), although noticeable sample-to-sample variance (Supplementary Figure 8) is observed. In contrast, for $\varphi > \varphi_{\rm G}$, the stress-strain curve becomes realization-dependent. This observation is consistent with our basic expectation: the free energy landscape is complex in this regime, and the system could fall into different sub-basins after compression.

Relaxation of the shear stress: connection to the free-energy landscape

To better understand the relaxation of shear stress upon a instantaneous shear strain γ, in particular the behavior of $\sigma_{\rm ZFC}(\tau, t_{\rm w})$ in the Gardner phase (Fig. 2b in the main text), for comparison we schematically plot $\sigma_{\rm ZFC}(\tau, t_{\rm w})$ anticipated from a theoretical point of view [3] (Supplementary Figure 9). The key feature is that, after an initial fast decay within the ballistic time scale $\tau_{\rm b}$, two steps of relaxations are expected: the $\sigma_{\rm ZFC}(\tau, t_{\rm w})/\gamma$ firstly relaxes to the plateau corresponding to $\mu_{\rm ZFC}$ at time $\tau \sim \tau_\beta$, and then it further relaxes to the second plateau corresponding to $\mu_{\rm FC}$ at $\tau \sim \tau_{\rm mb}$. Here τ_β and $\tau_{\rm mb}$ are the times needed to explore a single glass sub-basin and a glass meta-basin respectively. At even larger time $\tau \sim \tau_\alpha$, the stress may eventually relaxes to zero due to α-processes. In our study, the initial configurations at $\varphi_{\rm g}$ are deeply equilibrated, such that τ_α is far beyond our simulation time scale. Thus the α-relaxation is irrelevant in our analysis. However, if the initial configurations are far away from equilibrium, then τ_α could be comparable to the simulation time scale. In that case, the last step of relaxation towards zero stress may be observed [5, 6]. On the other hand, the behavior of $\sigma_{\rm FC}(t)$ is much simpler. As a one parameter function, it is $t_{\rm w}$-independent by definition, and $\sigma_{\rm FC}(t)/\gamma$ should converge quickly to $\mu_{\rm FC}$ after the initial ballistic processes.

Relaxation of the shear stress: the case of slow switching on of the shear strain

In the main text we discussed the relaxation of the stress after instataneous shear. Here let us examine how the shear stress relaxes if a small shear strain γ is applied *quasi-statically* (Supplementary Figure 10). The data should be compared with those in Fig. 2, where an instantaneous shear strain is applied. For $\varphi < \varphi_{\rm G}$, we do not see aging effects within our numerical accuracy. The zero-field compression (ZFC) and the field compression (FC) shear stresses converge quickly to the same value. For $\varphi > \varphi_{\rm G}$, no converge is observed within our simulation time window. The $\sigma_{\rm ZFC}(\tau, t_{\rm w})$ displays a plateau for short τ, followed by slow decay. Note that the time scale $\tau = 1$ used in determining $\mu_{\rm ZFC}$ (see the main text) is in the plateau region.

Time evolution of the pressure

In the main text, we plot the rescaled shear stress $\tilde{\sigma} = \sigma/p$ in Figs. 2 and 3, since a simple scaling relation $\sigma \sim p$ is expected in the normal glass phase. Here we examine whether the pressure p depends on time and protocol. Indeed, Supplementary Figure 11 shows that, in contrast to the stress, the pressure is nearly time-independent and protocol-independent after instantaneous shear, both below and above the Gardner transition. Therefore, $\tilde{\sigma}$ truly reveal the behavior of stress since we can treat p as a constant at any φ.

SUPPLEMENTARY NOTE 4 – ADDITIONAL DATA ON THE PROTOCOL-DEPENDENT SHEAR MODULUS

Here we report supplementary data on the protocol-dependent shear modulus. We discuss how the ZFC and the FC shear moduli depend on the the shear strain γ, the number of particles N, equilibrium density $\varphi_{\rm g}$, and the waiting time $t_{\rm w}$. We also measure the shear modulus using a third protocol.

Dependence on the shear strain γ

In our analysis, the shear modulus is measured by taking the ratio between the stress and the strain, i.e., $\mu_{\rm ZFC} = (\sigma_{\rm ZFC} - \sigma_0)/\gamma$ and $\mu_{\rm FC} = (\sigma_{\rm FC} - \sigma_0)/\gamma$, where σ_0 is the remanent shear stress. If γ is sufficiently small such that the non-linear corrections are negligible, the measured modulus should be independent of γ. Our data show that the FC modulus $\mu_{\rm FC}$ is indeed in such a linear regime for the chosen γ (see Fig. 3b in the main text for $N = 1000$ systems, and Supplementary Figure 12 for $N = 500$ and $N = 2000$ systems). However, γ-dependence is observed for $\mu_{\rm ZFC}$: at large pressure p close to jamming, $\mu_{\rm ZFC}$ increases for smaller shear strain γ. For smaller γ, the large-p scaling $\mu_{\rm ZFC} \sim p^{\kappa}$ agrees better with the mean-field theory, for any N studied, but additional data are required to conclude if the mean-field result is coincided in the limit $\gamma \to 0$. Recently, a very careful study shows that the mean-field jamming exponents, which characterize the critical distribution of small inter-particle gaps and weak contact forces, are consistent with simulation data in finite dimensions, after removing localized bucking excitations [7]. Such an analysis in the $p \to \infty$ limit is beyond the present numerical accuracy.

Dependence on the number of particles N

For a fixed γ, Fig. 3 in the main text shows that at large p, $\mu_{\rm ZFC}$ decreases with increasing N. It suggests that the non-linear effect, associated to stress relaxation due to MPEs, is stronger in larger systems. Indeed, in Ref. [8], the authors found a finite-size scaling $\delta\gamma_1 \sim N^{\beta}$ with $\beta \approx -0.62$, for the mean strain $\delta\gamma_1$ at which the first MPE takes place in amorphous solids. This scaling suggests that, MPEs are easier to occur in larger systems, and become unavoidable at any finite shear strain in the thermodynamic limit, because $\delta\gamma_1 \to 0$ as $N \to \infty$. It is thus reasonable to see that data with smaller γ (for a fixed N) or smaller N (for a fixed finite γ) obeys better the mean-field scaling $\mu_{\rm ZFC} \sim p^{1.41574}$, because the theoretical $\mu_{\rm ZFC}$ is only concerned about the linear response [3].

In our data (Fig. 3b in the main text and Supplementary Figure 12), we do not find appreciable N-dependence of $\mu_{\rm FC}$. In contrast, a scaling relation $\mu_{\rm FC} \sim N^{-0.25}$ was reported in Ref. [5]. Here we discuss possible reasons for this discrepancy. In [5], the systems are quenched from completely random initial configurations. Compared to the case in well equilibrated systems, in such non-equilibrium systems, the stress relaxes much faster and eventually decays to zero, i.e., the system melts quickly [6] (see Supplementary Figure 9 for an illustration). Considering that larger

systems have an easier tendency to relax, we expect that in the thermodynamic limit, the system turns to a liquid within the simulation time scale used in Ref. [5], which is the reason why $\mu_{\mathrm{FC}} \to 0$.

Dependence on the initial equilibrium density φ_{g}

We find that our basic observation – the bifurcation between the ZFC shear modulus μ_{ZFC} and the FC shear modulus μ_{FC} at the Gardner transition φ_{G} – is independent of the initial equilibrium density φ_{g} (see Supplementary Figure 13). Note that the value of φ_{G} itself depends on φ_{g}. The large pressure ($\varphi \gg \varphi_{\mathrm{G}}$) scalings, μ_{ZFC} versus p, and μ_{FC} versus p, are nearly unchanged for different φ_{g}. Additionally, we compare our simulation data with theoretical predictions for $\varphi < \varphi_{\mathrm{G}}$. We plot $\tilde{\mu}_{\mathrm{ZFC}}/\tilde{\mu}_{\mathrm{g}}$ and $\tilde{\mu}_{\mathrm{FC}}/\tilde{\mu}_{\mathrm{g}}$ as functions of p/p_{g} obtained from simulations, together with the mean-field *state following* theory [2], where $\tilde{\mu} = \mu/p$ is the modulus rescaled by the pressure, and μ_{g} and p_{g} are the shear modulus and the pressure at φ_{g}. Note that the theory does not distinguish between ZFC and FC moduli in this regime. On this rescaled plot, the theory and the simulation data show similar behaviors, both of which are insensitive to φ_{g}. We point out that the mean-field theory uses an over-simplified liquid EOS, that is only valid for mono-disperse hard spheres in the large dimensional limit. Thus a direct comparison between the theory and our simulation is impossible. However, once the effect of this mismatch on the liquid structure is removed by a proper rescaling with respect to the reference point at φ_{g}, the theory basically captures the general trend on how the system evolves under a slow compression annealing.

Dependence on the waiting time t_{w}

In Supplementary Note 3, we have discussed how $\sigma_{\mathrm{ZFC}}(\tau, t_{\mathrm{w}})$ and $\sigma_{\mathrm{FC}}(\tau, t_{\mathrm{w}})$ relax with τ under a quasi-static shear strain γ. Based on the data (Supplementary Figure 10), we choose time scales $\tau = 1$ and $\tau_{\mathrm{w}} = 10$ to measure μ_{ZFC} and μ_{FC} (see the main text). The scale $\tau = 1$ is chosen within the first plateau regime of $\sigma_{\mathrm{ZFC}}(\tau, t_{\mathrm{w}})$. Note that for larger τ, the difference $\mu_{\mathrm{ZFC}} - \mu_{\mathrm{FC}}$ would decrease, and would eventually vanish as $\tau \to \infty$, even in the Gardner phase. On the other hand, in order to examine the t_{w}-dependence more carefully, we obtain additional data of μ_{ZFC} and μ_{FC} for a few different t_{w} (we fix $\tau = 1$ in all cases). Interestingly, we found that the differences $\mu_{\mathrm{ZFC}} - \mu_{\mathrm{FC}}$ obtained by using different t_{w}, when plotted as a function of φ, collapse onto the same curve, within our numerical accuracy (Supplementary Figure 14). In particular, because the Gardner transition is determined as the bifurcation point between μ_{ZFC} and μ_{FC}, this result shows that the location of the transition point is independent of t_{w} in our approach.

A third protocol

To further verify and emphasize the protocol dependence on the shear modulus, we design a third protocol, in which we apply an additional shear strain after the FC procedure. In this protocol, we first apply a small quasi-static strain γ at φ_{g}, compress the system to φ, and then after waiting for $t_{\mathrm{w}} = 10$ and $\tau = 1$, we measure the stress σ_{FC}. This procedure is basically the same as the FC (see the main text). We then apply an additional quasi-static strain γ at φ, and measure the stress σ_{FCS} after waiting for $\tau = 1$. The FCS (FC+shear) modulus is defined as $\mu_{\mathrm{FCS}} = (\sigma_{\mathrm{FCS}} - \sigma_{\mathrm{FC}})/\gamma$. Supplementary Figure 15 shows that this shear modulus is close to μ_{ZFC}, and clearly different from μ_{FC}. From the view point of free energy landscape, for $\varphi > \varphi_{\mathrm{G}}$, μ_{FCS} represents the local curvature of the sub-basins at a finite given γ (see Fig. 4 in the main text), while μ_{ZFC} is the local curvature of the sub-basins at $\gamma = 0$.

SUPPLEMENTARY NOTE 5 – PROTOCOL-DEPENDENT SHEAR MODULUS OF BIDISPERSE HARD DISKS

To test if the ZFC/FC approach can be applied to other systems, we also study a two-dimensional bidisperse hard disk model glass former. The system contains $N = 1000$ equimolar bidisperse hard disks with diameter ratio $D_1 : D_2 = 1.4 : 1$. The dynamical crossover density is $\varphi_{\mathrm{d}} = 0.790(1)$ [1]. The example in Supplementary Figure 16 shows that the shear modulus becomes protocol-dependent for $\varphi > \varphi_{\mathrm{G}}$. This signature can be used to determine φ_{G}, whose value is fully consistent with the previous independent estimate [1].

SUPPLEMENTARY METHODS – NUMERICAL PROTOCOLS

Compression protocols

Generation of dense equilibrium liquids – To prepare dense equilibrium configurations, we combine the Lubachevsky-Stillinger (LS) algorithm [9] with swap Monte Carlo moves [1, 4]. The LS algorithm consists of standard event-driven molecular dynamics (MD), and slow compression which is realized by variation of the diameter of particles. Our protocol consists of the following two steps:

1. Starting from an ideal gas configuration, we first compress it to $\varphi_0 = 0.54$, by growing spheres at a constant rate $\delta_{\rm g} = 10^{-3}$, such that $D(t) = D(0)(1 + \delta_{\rm g} t)$. Because the process is equivalent to compression, hereafter we call $\delta_{\rm g}$ as compression rate. This initial compression is fast enough to suppress crystallization, and slow enough to equilibrate the configuration up to φ_0.

2. Starting from the equilibrium configuration at φ_0, we switch to a slower compression rate $\delta_{\rm g} = 10^{-5}$, and compress the configuration to a higher density $\varphi_{\rm g}$. Swap attempts are introduced: we randomly pick a pair of particles and exchange their diameters if no overlap is created after the swap. The particle sizes do not change during swap moves. We perform 10% swap moves and 90% LS molecular dynamics steps. After the compression, we further relax the configuration for $t = 1000$ to check that the pressure does not change. The equation of state (EOS) of our equilibrium configurations agrees with the Carnahan-Stirling (CS) expression (see Supplementary Figure 1) [10],

$$p_{\rm CS}(\varphi) = \frac{1}{1-\varphi} + \frac{3 s_1 s_2}{s_3} \frac{\varphi}{(1-\varphi)^2} + \frac{s_2^3}{s_3^2} \frac{(3-\varphi)\varphi^2}{(1-\varphi)^3}, \qquad \text{(Supplementary Equation 4)}$$

   where s_k is the k-th moments of the diameter distribution function $P(D)$.

Generation of glasses – The swap algorithm is swithced off once the equilbriated configuration at the target $\varphi_{\rm g}$ is obtained. All the subsequent simulations are performed using the MD without swap. Since the α-relaxation time has become much larger than our MD simulation time scales, we are left with a piece of glass. We call the configuration of the particle positions $\{\mathbf{r}_i\}$ $(i = 1, 2, \ldots, N)$ of such a glass at $\varphi_{\rm g}$ as a *sample*. From each of such a sample at $\varphi_{\rm g}$, we generate many *realizations* by setting random particle velocities $\{\mathbf{v}_i\}$ $(i = 1, 2, \ldots, N)$ drawn from the Maxwell-Boltzmann disribution. Each of such realizations is compressed by the LS algorithm to obtain compressed glasses at desired densities $\varphi > \varphi_{\rm g}$. Note that the kinetic energy is conserved so that the system remains at the unit temperature throughout our simulations.

Shear protocols

Quasi-static shear – In the quasi-static shear, the shear strain γ is increased with time at a constant rate $\dot{\gamma}$, which is set small enough such that the system is quasi-equilibrated at each step. The protocol consists of the following steps:

1. Increase the shear strain γ instantaneously by an infinitesimal amount $\gamma \to \gamma + \delta\gamma$ with $\delta\gamma = 10^{-4}$. We perform an affine deformation to all particles, whose positions are shifted by $x_i \to x_i + \delta\gamma z_i$, where x_i and z_i are the $x-$ and $z-$coordinates of particles i. This instantaneous shift could introduce overlaps between some particles, which are removed by using the the conjugated gradient (CG) method [11]. To use CG, a harmonic inter-particle potential $\phi_{ij}(r) = (1 - r/D_{ij})^2$ (zero when $r > D_{ij}$) is used, where $D_{ij} = (D_i + D_j)/2$ is the average diameter of particles i and j. The boundary condition in the z direction satisfies the Lees-Edwards scheme [12], i.e., the x-position of the top (bottom) imaginary box is shifted by $\delta\gamma L$ $(-\delta\gamma L)$, where L is the linear size of the simulation box. After this step, we obtain an non-overlapping hard sphere (HS) configuration under shear strain $\gamma + \delta\gamma$.

2. We switch the soft potential back to the hardcore potential, and equilibrate the system by using the event-driven MD to simulate the dynamics of HSs under fixed shear strain $\gamma + \delta\gamma$. Again we emphasize that the dynamics preserves the kinetic energy so that the system remains at a constant temperature. The velocities are reinitialized after each step of the shear strain. The Lees-Edwards boundary condition is kept. We perform LS simulation for a duration δt, such that $\delta\gamma/\delta t = \dot{\gamma}$.

3. The above two steps are repeated until the shear strain reaches a target value.

To simulate quasi-static shear, we choose a sufficiently small $\dot{\gamma} = 10^{-4}$. We have checked that for $\varphi < \varphi_{\rm G}$, the stress-strain response is independent of $\dot{\gamma}$ when $\dot{\gamma}$ is decreased from 10^{-3} to 10^{-5} (see Supplementary Note 3).

Instantaneous shear – To simulate instantaneous shear, we instantaneously increase the shear strain from 0 to γ. We then turn on the harmonic soft-potential, and use the CG algorithm to remove the overlaps. Different from the quasi-static shear, the system is generally far away from equilibrium after the instantaneous shear.

[1] Ludovic Berthier, Patrick Charbonneau, Yuliang Jin, Giorgio Parisi, Beatriz Seoane, and Francesco Zamponi, "Growing timescales and lengthscales characterizing vibrations of amorphous solids," Proc. Nat. Acad. Sci. U.S.A. **113**, 8397–8401 (2016).

[2] Corrado Rainone, Pierfrancesco Urbani, Hajime Yoshino, and Francesco Zamponi, "Following the evolution of hard sphere glasses in infinite dimensions under external perturbations: Compression and shear strain," Phys. Rev. Lett. **114**, 015701 (2015).

[3] Hajime Yoshino and Francesco Zamponi, "Shear modulus of glasses: Results from the full replica-symmetry-breaking solution," Phys. Rev. E **90**, 022302 (2014).

[4] Ludovic Berthier, Daniele Coslovich, Andrea Ninarello, and Misaki Ozawa, "Equilibrium sampling of hard spheres up to the jamming density and beyond," Phys. Rev. Lett. **116**, 238002 (2016).

[5] Daiju Nakayama, Hajime Yoshino, and Francesco Zamponi, "Protocol-dependent shear modulus of amorphous solids," J. Stat. Mech. **2016**, 104001 (2016).

[6] Satoshi Okamura and Hajime Yoshino, "Rigidity of thermalized soft repulsive spheres around the jamming point," arXiv:1306.2777 (2013).

[7] Patrick Charbonneau, Eric I. Corwin, Giorgio Parisi, and Francesco Zamponi, "Jamming criticality revealed by removing localized buckling excitations," Phys. Rev. Lett. **114**, 125504 (2015).

[8] Smarajit Karmakar, Edan Lerner, and Itamar Procaccia, "Statistical physics of the yielding transition in amorphous solids," Phys. Rev. E **82**, 055103 (2010).

[9] Monica Skoge, Aleksandar Donev, Frank H. Stillinger, and Salvatore Torquato, "Packing hyperspheres in high-dimensional Euclidean spaces," Phys. Rev. E **74**, 041127 (2006).

[10] T. Boublik, "Hard sphere equation of state," J. Chem. Phys. **53**, 471 (1970).

[11] William H Press, *Numerical recipes 3rd edition: The art of scientific computing* (Cambridge university press, 2007).

[12] AW Lees and SF Edwards, "The computer study of transport processes under extreme conditions," J. Phys. Condens. Matter **5**, 1921 (1972).
